# Pioneer transcription factors in cell reprogramming

**DOI:** 10.1101/gad.253443.114

**Published:** 2014-12-15

**Authors:** Makiko Iwafuchi-Doi, Kenneth S. Zaret

**Affiliations:** Institute for Regenerative Medicine, Department of Cell and Developmental Biology, Perelman School of Medicine, University of Pennsylvania, Philadelphia, Pennsylvania 19104, USA

**Keywords:** pioneer transcription factor, reprogramming, nucleosome, chromatin, transdifferentiation, development

## Abstract

A subset of eukaryotic transcription factors possesses the remarkable ability to reprogram one type of cell into another. The transcription factors that reprogram cell fate are invariably those that are crucial for the initial cell programming in embryonic development. To elicit cell programming or reprogramming, transcription factors must be able to engage genes that are developmentally silenced and inappropriate for expression in the original cell. Developmentally silenced genes are typically embedded in “closed” chromatin that is covered by nucleosomes and not hypersensitive to nuclease probes such as DNase I. Biochemical and genomic studies have shown that transcription factors with the highest reprogramming activity often have the special ability to engage their target sites on nucleosomal DNA, thus behaving as “pioneer factors” to initiate events in closed chromatin. Other reprogramming factors appear dependent on pioneer factors for engaging nucleosomes and closed chromatin. However, certain genomic domains in which nucleosomes are occluded by higher-order chromatin structures, such as in heterochromatin, are resistant to pioneer factor binding. Understanding the means by which pioneer factors can engage closed chromatin and how heterochromatin can prevent such binding promises to advance our ability to reprogram cell fates at will and is the topic of this review.

## Abstract (precis)

Biochemical and genomic studies have shown that transcription factors with the highest reprogramming activity often have the special ability to engage their target sites on nucleosomal DNA, thus behaving as “pioneer factors” to initiate events in closed chromatin. This review by Iwafuchi-Doi and Zaret focuses on the most recent studies of pioneer factors in cell programming and reprogramming, how pioneer factors have special chromatin-binding properties, and facilitators and impediments to chromatin binding.

Cell fate control represents the most extreme form of gene regulation. Genes specific to the function of a particular type of cell may be antagonistic to the function of another type of cell, so nature has evolved diverse regulatory mechanisms to ensure stable patterns of gene activation and repression. In prokaryotes, self-sustaining regulatory networks of transcription factors bound to DNA can be sufficient to regulate gene activation and repression (Ptashne 2011). In eukaryotes, ∼200-base-pair (bp) segments of the genome are wound nearly twice around an octamer of the four core histones to form nucleosomes, providing steric constraints on how transcription factors can bind DNA (Kornberg and Lorch 1999). As described below, pioneer transcription factors have the special property of being able to overcome such constraints, enabling the factors to engage closed chromatin that is not accessible by other types of transcription factors (Fig. 1). However, further higher-order packaging of nucleosomes into heterochromatin can occlude access to DNA altogether, presenting an additional barrier to cell fate conversion (Fig. 1). This review focuses on the most recent studies of pioneer factors in cell programming and reprogramming, how pioneer factors have special chromatin-binding properties, and facilitators and impediments to chromatin binding. We project that such knowledge will greatly aid future efforts to change the fates of cells at will for research, diagnostic, and therapeutic purposes.

**Figure 1.**
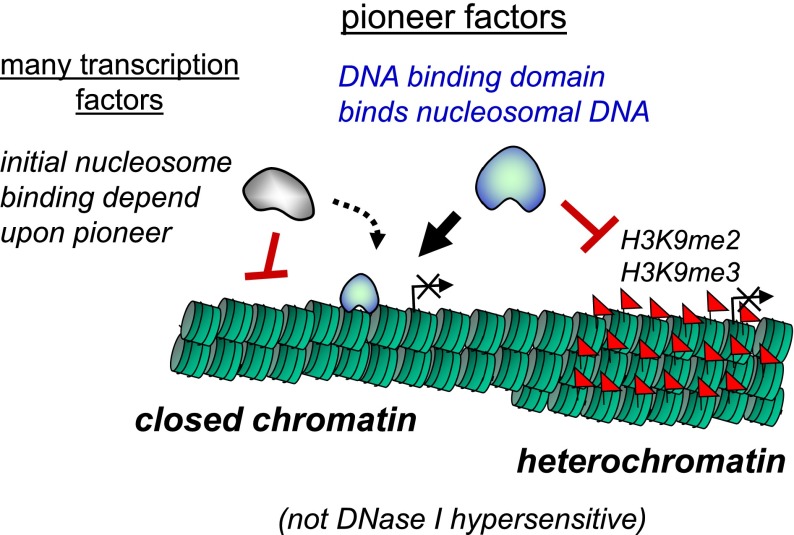
Initial targeting of closed chromatin by pioneer factors. The DNA-binding domain of pioneer factors allows the protein to recognize its target site on nucleosomal DNA. The initial targeting of nucleosomal DNA by pioneer factors occurs in closed, silent chromatin that lacks nuclease hypersensitivity and consistent histone modifications or other prior marks. This allows the pioneer factor to initiate reprogramming of silent genes that may be inappropriate to express in a given cell, enabling cell type conversion. Many transcription factors (nonpioneers) cannot initially target such genes but can do so coordinately with, or after, pioneer factors bind. However, certain heterochromatic regions of the genome, such as where H3K9me2 or H3K9me3 marks are deposited, are refractory to pioneer factor binding. Continuing research on how pioneer factors can target nucleosomal sites and how heterochromatic impediments can be broken down will inform ways to enhance our ability to control cell fates for biomedical purposes.

## Who’s on first?

Which transcription factors are the first to access a developmentally silent gene and initiate its expression to promote a cell fate change? Genetic studies alone cannot answer this, as a transcription factor may appear necessary or sufficient to elicit a regulatory change but dependent on the prior binding of other factors in the cell. Why does knowing “who’s on first” matter? Because the hierarchical mechanisms by which transcription factors engage target sites in chromatin provide insights into ways to modulate the process and hence cell fate control.

**Figure 2.**
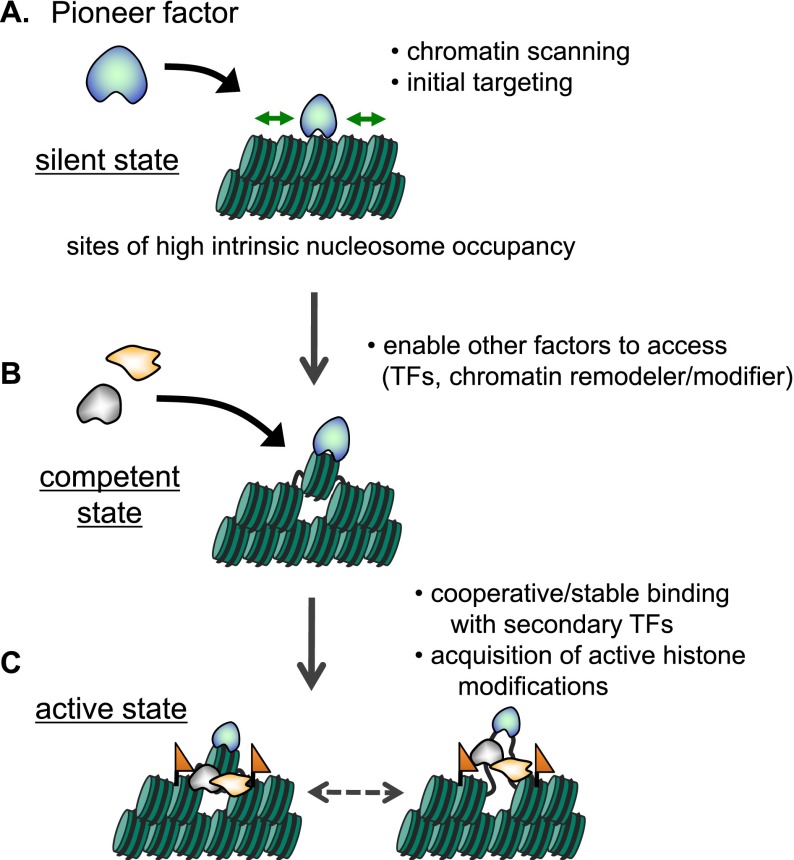
Initial targeting of pioneer factor and subsequent events. (*A*) Pioneer transcription factors can target sites with high intrinsic nucleosome occupancy. (*B*) Pioneer factors initially engage nucleosomal target sites, which enable other factors (transcription factors, chromatin modifiers, and remodelers) to access the target sites. (*C*) Other transcription factor (TF)-binding and chromatin modifications could stabilize pioneer factor binding to the target sites.

In diverse contexts where groups of transcription factors have been tested for their ability to convert cell fate, a subset of factors consistently has the greatest effect in cell conversion. Biochemical and genomic studies indicate that such factors can be considered “pioneers” by virtue of their ability to engage target DNA sites in closed chromatin prior to the apparent engagement, opening, or modification of the site by other factors (Box 1). Nucleosome binding by pioneer factors typically enables the coordinate or subsequent binding of other transcription factors, cofactors, and chromatin-modifying and remodeling enzymes, culminating in the activation of genes of a new cell fate (Fig. 2). The main point is that the nucleosome-binding activity of pioneer factors allows them to initiate regulatory events at particular sites in chromatin that have not been programmed for expression, as typically seen in developmentally silent genes.

**Box 1. Features of pioneer transcription factors**

Engage their target sites in closed (nuclease-resistant), silent chromatin prior to gene activity.
Increase accessibility of a target site that makes other proteins (e.g., transcription factors, chromatin remodelers, chromatin modifiers, histone variants, and repressors) accessible to the site.
Play a primary role in cell programming and reprogramming and establish the competence for cell fate changes.

**How to predict/validate pioneer factors**

Observe the chromatin state of target sites for a transcription factor before and after the factor is expressed in a cell. Pioneer factors can target sites that are closed (nuclease-resistant; e.g., with ATAC-seq and DNase-seq) or shown to be nucleosomal (MNase-Seq) prior to binding and often result in chromatin opening upon binding. Merely correlating open chromatin at sites where a transcription factor is already bound does not predict pioneer factors.
Analyze direct binding between a transcription factor and a reconstituted mononucleosome or a nucleosomal array in vitro (e.g., with electrophoretic mobility shift assays, DNase I footprinting, and sequential transcription factor and core histone ChIP).
Analyze the effect of a transcription factor binding on DNA accessibility at the target sites in vitro (reconstituted nucleosomal array with a transcription factor) or in vivo (ectopic expression or deletion of a transcription factor).

We start by reviewing the latest genome-mapping studies of chromatin states before and after transcription factor engagement in the contexts of development and cell reprogramming, which provide evidence that pioneer factors have special chromatin-binding properties suitable for their pioneering function. We then review the molecular mechanisms that underlie chromatin binding, the limits of such binding, and the importance of overcoming chromatin impediments in order to control cell fate.

## Pioneer factors in zygotic genome activation

Maternal factors in the oocyte trigger zygotic genome activation, which is perhaps the most dramatic reprogramming event in embryogenesis (Tadros and Lipshitz 2009). The maternal transcription factor Zelda (Zld; zinc finger early *Drosophila* activator) plays a primary role for the onset of zygotic genome activation in *Drosophila* embryos (Liang et al. 2008; Nien et al. 2011). Zld protein is present in nuclei considerably earlier than other key maternal transcription factors such as Bicoid (Bcd) and Dorsal (Dl) proteins and is bound to gene regulatory regions prior to zygotic genome activation (Harrison et al. 2011; Nien et al. 2011). Zld binding increases DNA accessibility and facilitates the binding of other transcription factors, including Bcd and Dl, to target enhancers (Foo et al. 2014; Xu et al. 2014). Furthermore, differential DNA accessibility established by different levels of Zld binding sets the threshold for responding to the Dl gradient: More open enhancers are activated even where Dl concentration is low, but fewer open enhancers are activated only where Dl concentration is high (Foo et al. 2014). While in vitro nucleosome-binding studies have yet to be reported for Zld, by all in vivo criteria, Zld appears to function as a pioneer factor for zygotic genome activation.

Although homologs of Zld have not been reported outside the insect clade, in zebrafish, Nanog, Pou5f3 (originally named Pou2 and Pou5f1; a member of the class V POU family, as is mammalian Oct3/4), and the functionally redundant SoxB1 group of transcription factors (Sox2, Sox3, Sox19a, and Sox19b) are highly enriched and bound to their target sites prior to zygotic genome activation. They also play primary roles in the onset of zygotic genome activation (Lee et al. 2013; Leichsenring et al. 2013). In mice, maternal Oct3/4 and Sox2 are also primary regulators of zygotic genome activation (Foygel et al. 2008; Pan and Schultz 2011). Altogether, factors that activate the zygotic genome can engage their target sites in chromatin that is not preprogrammed, can elicit local chromatin changes, and can enable subsequent gene expression, thus having the hallmarks of pioneer transcription factors (Table 1).

**Table 1.**
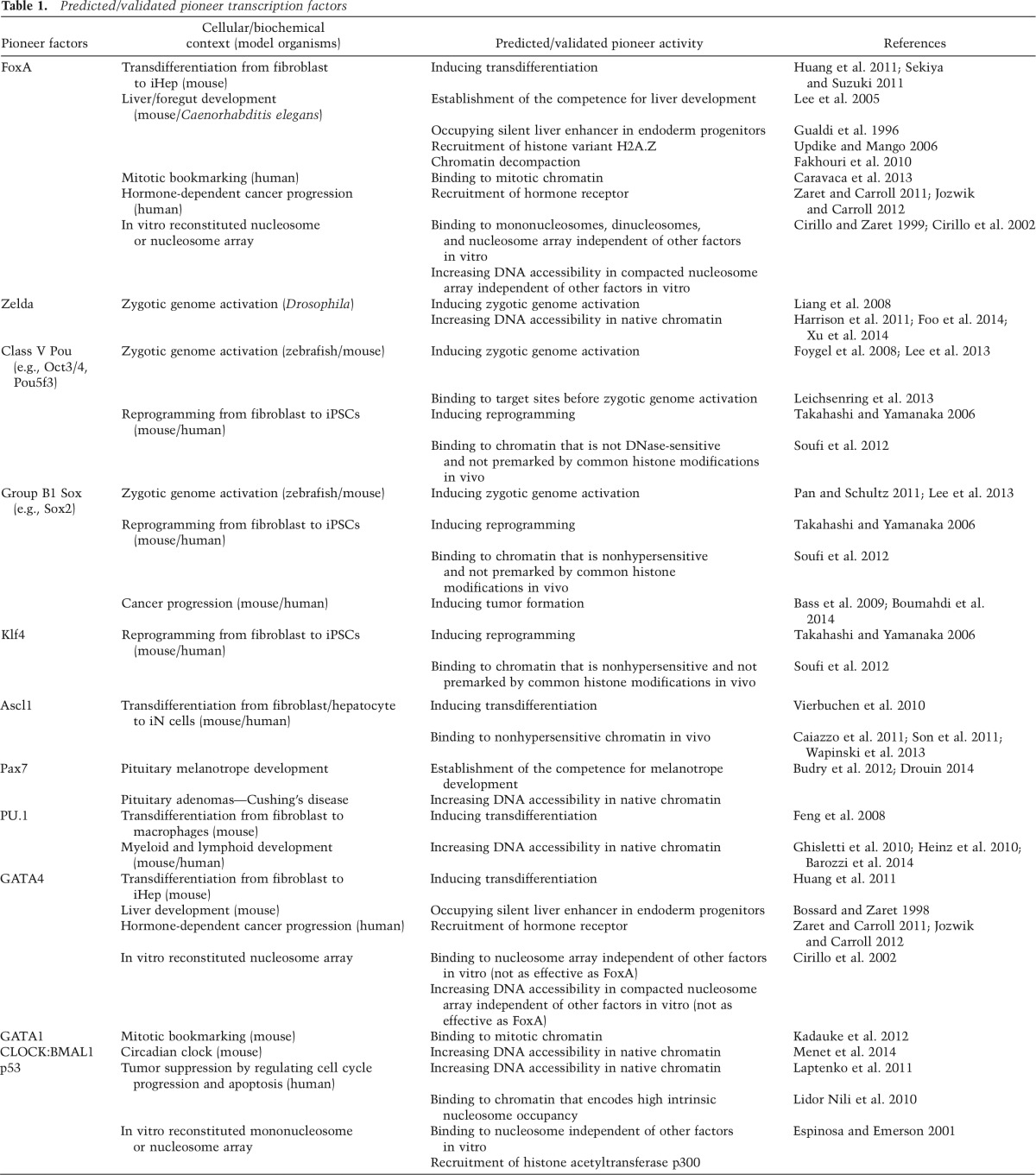
Predicted/validated pioneer transcription factors

## Pioneer factors in cell reprogramming

Reprogramming of terminally differentiated cells was first shown by somatic cell nuclear transfer into enucleated oocytes (Gurdon 1962), indicating that factors in the oocyte cytoplasm can reprogram somatic nuclei to a pluripotent state. By screening diverse factors that are normally expressed in pluripotent stem cells (PSCs), but not fibroblasts, for their ability to convert fibroblasts to pluripotency, the transcription factors Oct3/4, Sox2, Klf4, and c-Myc (O, S, K, and M) were found to trigger endogenous expression of pluripotent factors and be sufficient to reprogram fibroblasts into induced PSCs (iPSCs) (Takahashi and Yamanaka 2006). Although alternative sets of transcription factors for iPSC reprogramming have been reported (e.g., Buganim et al. 2014), most studies include Oct3/4 and/or Sox2 (Yu et al. 2007; Feng et al. 2009; Han et al. 2010; Gao et al. 2013). How does this set of factors first interact with their target sites to initiate reprogramming? A snapshot of the initial binding events of OSKM in human fibroblasts indicates their preferential occupancy of promoter-distal (i.e., enhancer) target sites (Soufi et al. 2012). Many initial binding events occur at genes that elicit reprogramming to pluripotency as well as at genes that promote apoptosis during the early stages of iPSC reprogramming. Many more initial binding events are distinct from the definitive binding pattern in embryonic stem (ES) cells or iPSCs that maintains pluripontency (Soufi et al. 2012). Thus, the initial binding or scanning of the genome is quite promiscuous during reprogramming of somatic cells to pluripotency, and subsequent reorganization of the factors in the genome must occur to establish the final pluripotent state (Hochedlinger and Plath 2009).

Of these factors, Oct3/4, Sox2, and Klf4 act as pioneer transcription factors in that they can access closed chromatin whether they bind together or alone (Fig. 1; Table 1; Soufi et al. 2012). “Closed chromatin” in this context means to lack DNase hypersensitivity and a lack of a consistent histone modification pattern. In contrast, c-Myc alone prefers to bind to “open chromatin” sites that are DNase-hypersensitive and contain activating histone modifications (Soufi et al. 2012). However, c-Myc can bind closed chromatin sites in conjunction with the other factors (Soufi et al. 2012). These findings illustrate how a cohort of factors, all of which are necessary in genetic tests of efficient conversion to pluripotency, can differ markedly with regard to whether they are pioneer factors. In addition, the pioneer factors can directly enable nonpioneers to engage closed chromatin sites. The exact mechanisms by which such facilitation occurs remain to be determined but could include interactions between the pioneer factors and the nucleosomes that enable other factors to bind, direct interactions between the factors themselves, and/or recruitment of cofactors or nucleosome remodelers that in turn alter the local chromatin landscape to facilitate secondary factor binding (Fig. 2).

Single-molecule tracking studies in live cells suggested that during iPSC reprogramming, Sox2 is bound to the target DNA first, followed by binding of Oct3/4 to stabilize the ternary complex (Chen et al. 2014). These live-cell results do not match the ChIP-seq (chromatin immunoprecipitation [ChIP] combined with deep sequencing) data, where the majority of the initial Oct3/4- and Sox2-binding events were found to be independent of one another (Soufi et al. 2012). The ChIP-seq studies with native OSKM proteins were performed under conditions where continued culture of the OSKM-induced cells led to iPSC conversion. In contrast, the single-molecule studies required apparently higher ectopic expression of HALO-tagged factors, which covalently bind a fluorescent ligand in short growth period experiments. It could be that the single-molecule studies were revealing chromatin state-dependent binding for the factors to engage DNase-sensitive, apparently free DNA sites in the nucleus, which represented a minority of events in the ChIP-seq studies. We need direct nucleosome- and chromatin-binding studies to assess exactly how the OSKM factors mechanistically and sequentially engage chromatin to initiate the dramatic reprogramming to pluripotency. That the Pou5f3 and Sox2 factors can serve a pioneering function during zygotic genome activation (see above) underscores the special abilities of these factors.

## Pioneer factors in direct cell conversion (transdifferentiation)

Direct cell conversion (transdifferentiation) by individual transcription factors was shown for the first time when MyoD was found to induce muscle-specific properties when ectopically expressed in fibroblasts (Davis et al. 1987). However, exogenous MyoD in endodermal and ectodermal cells failed to induce a full phenotypic switch (Weintraub et al. 1989; Schäfer et al. 1990). Later studies revealed that MyoD induces *myogenin* through cooperation with the Pbx factor, which is constitutively bound to MyoD target sites in fibroblasts prior to MyoD expression (Berkes et al. 2004). There are many other examples of direct cell conversion within the same germ layer: the combination of PU.1 and C/EBPα into macrophage-like cells from fibroblasts (Feng et al. 2008) and GATA4, MEF2C, TBX5, Hand2, and NKX2.5 for cardiomyocyte-like cells from fibroblasts (Ieda et al. 2010; Addis et al. 2013).

The first evidence of efficient transdifferentiation across different germ layers was the generation of functional glutaminergic neurons (inducible neurons [iNs]) from fibroblasts by the three transcription factors Ascl1, Brn2, and Myt1l (Vierbuchen et al. 2010). Of these factors, Ascl1 plays a central role to initiate transdifferentiation because Ascl1 alone is sufficient to induce immature iN cells, but Brn2 and Myt1l are not. Soon after, dopaminergic and motor neurons were generated from fibroblasts using different sets of transcription factors, but both sets included Ascl1 (Caiazzo et al. 2011; Son et al. 2011). Recently, Wernig and colleagues (Wapinski et al. 2013) revealed a hierarchical mechanism governing the early stage of transdifferentiation to iNs: Ascl1 acts as a pioneer transcription factor to bind closed chromatin and recruit Brn2 to Ascl1 target sites (Table 1), and Brn2 is primarily required for the later stage of transdifferentiation by contributing to iN maturation. These experiments show that even when the factors are simultaneously overexpressed, they function in a hierarchical manner, as described above for Oct3/4, Sox2, Klf4, and c-Myc. Ascl1 acts first to establish competence for the neuronal lineage, followed by other transcription factors to specify neuronal subtypes. Like the nonpioneer c-Myc, Ascl1 is a basic helix–loop–helix (bHLH) family transcription factor whose core structure is thought to bind to both sides of the DNA helix (Bertrand et al. 2002). However, unlike c-Myc, Ascl1 appears to have properties of a pioneer factor. It will be important to determine how different members of the bHLH class, which exhibit differences in how they contact DNA, can or cannot function as pioneer factors.

Furthermore, Ascl1 was characterized as an “on-target” pioneer factor in that most of its initially bound sites, when ectopically expressed in fibroblasts, correspond to sites that remain bound when the cells finally differentiate into neurons (Wapinski et al. 2013). This in contrast to Oct3/4, Sox2, and Klf4 initially binding to the genome in fibroblasts, where, as described above, many of the initial binding events do not correspond to those after full conversion to iPSCs (Soufi et al. 2012). This could reflect inherent differences in how Ascl1 versus Oct3/4, Sox2, and Klf4 initially recognize their target sites in chromatin.

Alternatively, there could be an inherent difference in the overall chromatin structure of pluripotent cells that takes multiple phases to recapitulate by reprogramming to iPSCs, whereas direct conversion among differentiated cells may not involve as dramatic an overall chromatin change. For example, fluorescence recovery after photobleaching experiments have suggested that pluripotent cell chromatin is more “hyperdynamic” than somatic cell chromatin, allowing for a more rapid exchange of chromatin components than in differentiated cells (Meshorer et al. 2006). Thus, global changes in chromatin structure may be necessary for reprogramming to iPSCs, and hence cells may have to go through various stages with different binding patterns at each stage.

Another example of transdifferentiation across germ layers is when hepatocyte-like cells are induced from fibroblasts (iHep) by exogenous expression of either forkhead box protein A1 (FoxA1), FoxA2, or FoxA3 with HNF4a (Sekiya and Suzuki 2011) or exogenous expression of FoxA3, GATA4, and HNF1a in combination with the inactivation of the tumor suppressor gene p19^Arf^ (Huang et al. 2011). These factors were discovered from pools of many transcription factors involved in liver development; the common component was FoxA. However, compared with primary hepatocytes, iHeps show significant differences in gene expression and are unable to fully rescue liver function in a transplantation model (Huang et al. 2011; Sekiya and Suzuki 2011). CellNet analysis, which is a bioinformatics tool to assess the fidelity of cell conversion, surprisingly revealed that iHeps induced by FoxA and HNF4a represent gut endoderm progenitors more than mature hepatocytes (Morris et al. 2014). They also show that iHeps are capable of engrafting both the mouse liver and colon, which are alternative fates from gut endoderm (Bort et al. 2006). It appears that the pioneer transcription factor FoxA and cofactors establish the competence to differentiate gut endodermal lineage from fibroblasts (Table 1), and additional lineage-determining factors are required for making mature hepatocytes. This model fits well with the original description of FoxA as endowing competence for the endoderm in embryos (Gualdi et al. 1996).

CellNet analyses further revealed that most transdifferentiation events fail to extinguish the expression programs of the starting cell type and thus impair the fidelity of cell type conversion (Cahan et al. 2014). Therefore, it is important to know both the hierarchical regulatory network required to activate a desired cell type and the means to extinguish the original gene regulatory program. This latter area appears to be a major gap in the field of cell type conversion. Along these lines, it would be important to know how to reset tissue-specific heterchromatic blocks that can be resistant to pioneer factor binding, as discussed below, and serve as an impediment to cell reprogramming (Soufi et al. 2012).

## Pioneer factors in cell programming during development

Not surprisingly, the transcription factors that have the strongest effect on cell reprogramming and transdifferentiation have been shown to play central roles in embryonic development. FoxA1 and FoxA2 are expressed in foregut endoderm and are required for the establishment of competence for liver development (Gualdi et al. 1996; Lee et al. 2005). GATA4 and GATA6 are expressed in foregut endoderm and are required for early liver development (Holtzinger and Evans 2005; Zhao et al. 2005; Watt et al. 2007). The liver-specific enhancer of the *albumin* (*Alb1*) gene contains six transcription factor-binding sites (McPherson et al. 1993). Of these, only binding sites for FoxA and GATA are occupied in the beginning of gut endoderm development, in which the *Alb1* gene has not yet been expressed (Gualdi et al. 1996; Bossard and Zaret 1998; Xu et al. 2012). Upon liver specification, all binding sites are occupied, and the *Alb1* gene becomes active. Thus, the FoxA and GATA factors act as pioneers for liver specification by engaging chromatin in multipotent progenitors and helping to provide the competence for hepatic fate (Table 1). During ES cell differentiation into endoderm in vitro, some FoxA2-binding sites are preoccupied by H2A.Z, and FoxA2 binding at the sites during endoderm induction results in nucleosome depletion and gene activation (Li et al. 2012a).

Hierarchical binding, in which pioneer factors bind first, is not unique to liver development. In pituitary melanotrope development, the initial expression of Pax7, a paired homeodomain transcription factor, slightly precedes the other melanotrope transcription factors, such as Tpit (Budry et al. 2012). Budry et al. (2012) exogenously expressed Pax7 in a pituitary corticotrope cell line in which Tpit is expressed but Pax7 is not. Exogenous Pax7 binding increased chromatin accessibility and induced de novo Tpit recruitment at the Pax7-binding sites. Pax7 is bound to DNA with either or both motifs for the protein’s paired domain and its homeodomain. Interestingly, Pax7 binding with both motifs gives more chromatin accessibility than those with a single motif. Although the intrinsic mechanism is not understood, Pax7 opens chromatin structure through the binding to composite motifs and provides competence for melanotrope development (Table 1; Drouin 2014).

PU.1 has been known to play a crucial role in myeloid and lymphoid development, and its expression there precedes other lineage-determining transcription factors (Carotta et al. 2010). PU.1 binding induces local nucleosome remodeling followed by the deposition of the active histone modification H3K4me1 (Heinz et al. 2010). PU.1 also engages other lineage-determining transcription factors (Heinz et al. 2010) and promotes overall nucleosome depletion (Barozzi et al. 2014). Additionally, PU.1 binding contributes to endotoxin-induced transcriptional activation in macrophages (Ghisletti et al. 2010). PU.1 is bound to a substantial fraction of the endotoxin-inducible enhancers prior to endotoxin stimulation to keep the enhancers accessible. Exogenous PU.1 expression in fibroblasts also induces accessible chromatin at de novo PU.1-binding sites in the inducible enhancers. PU.1 uses hydration to recognize target DNA and forms a long-lived complex relative to the other Ets factors, which use electrostatic interactions (Wang et al. 2014). We speculate that the hydration-based recognition imparts the necessary adaptability for PU.1 to bind nucleosomal DNA. Although more mechanistic studies are required to understand how PU.1 exerts its function, PU.1 can act as a pioneer factor to confer accessibility to the target enhancers during myeloid and lymphoid development (Table 1). Taken together, these studies show that pioneer factor binding occurs prior to lineage commitment and can employ a chromatin-opening step to establish the competence for gene activation.

Extensive computational analyses of changes in DNase cleavages across the genome have been studied during the in vitro differentiation of ES cells to mesendoderm, endoderm, prepancreatic, and intestinal endoderm fates (Sherwood et al. 2014). Sherwood et al. (2014) developed an elegant method called protein interaction quantitation (PIQ) that predicts transcription factor binding based on DNase footprinting and intrinsic binding motifs. PIQ then analyzes genome-wide correlations between a factor’s binding and the extent of local chromatin accessibility and thereby may predict pioneer transcription factors.

However, PIQ did not predict FoxA as a pioneer factor. As Sherwood et al. (2014) discussed, it could be because FoxA can recruit corepressors that create closed chromatin (Sekiya and Zaret 2007); such binding events by FoxA would not be scored because they would not be predicted to cause the hypersensitivity that is necessary for PIQ to identify pioneer activity. In addition, the PIQ method predicted only 50% of a factor’s actual binding sites detected by ChIP-seq.

Despite these issues, it is interesting that Sherwood et al. (2014) discovered that a subset of pioneer factors discerned by PIQ causes chromatin opening preferentially on one side of their nonpalindromic DNA-binding motif. That a cohort of factors behaved asymmetrically was striking and suggests directionally specific mechanisms to be discovered with regard to “downstream” events that follow pioneer factor binding.

Post-developmentally, there are many examples of where prior binding by FoxA factors enables the subsequent binding by hormone receptors and other DNA-binding proteins (Gao et al. 2003; Carroll et al. 2005; Zhang et al. 2005; Li et al. 2009, 2012b; Pihlajamaa et al. 2014), as previously covered in greater detail (Zaret and Carroll 2011). The mammalian circadian clock is driven by the CLOCK and BMAL1 transcription factors that rhythmically bind to nucleosomal target sites, promote incorporation of the histone variant H2A.Z, and enable the subsequent binding of other transcription factors (Menet et al. 2014). Thus, the mammalian clock appears to employ pioneer factors to activate genes at specific times of the daily cycle.

## The basis for pioneer activity: nucleosome binding and chromatin opening

What are the molecular mechanisms underlying pioneer function? The FoxA family of transcription factors has been examined in mechanistic depth by biochemical analyses on mononucleosomal, dinucleosomal, and nucleosomal array substrates in vitro in conjunction with in vivo footprinting (Table 1; Cirillo et al. 2002; Zaret and Carroll 2011). Purified FoxA protein can bind to its target sites on nucleosomal DNA, open a local domain of compacted chromatin, and stabilize nucleosome position (McPherson et al. 1996; Shim et al. 1998; Cirillo et al. 2002). Chromatin opening by FoxA in vitro does not require ATP or ATP-dependent chromatin remodelers (Cirillo et al. 2002). Notably, the DNA-binding domain of FoxA resembles that of linker histone and binds to one side of a DNA helix along the long axis of DNA, which leaves the other side of DNA to bind core histones (Clark et al. 1993; Ramakrishnan et al. 1993; Cirillo et al. 1998). Thus, the FoxA pioneer factor is structurally suited to recognize its DNA target sites on a nucleosome. Also, the C-terminal domain of FoxA can bind directly to core histone proteins and is required for opening chromatin (Cirillo et al. 2002). We need direct nucleosome-binding studies for other pioneer factors to determine how different DNA-binding domains may adapt to the nucleosome surface and how other domains of the proteins may interact directly with the core histones, perhaps destabilizing them. In addition, we need to understand how pioneer factors may engage chromatin-modifying enzymes to expand the “openness” of a local domain of chromatin, helping to activate an enhancer or a promoter.

## Nucleosomes at enhancers: a collaborator for pioneer factors

In higher eukaryotes, gene expression is regulated by the coordinated action of promoters, which determine a gene’s transcription start site, and one or more distal enhancers, which modulate promoter activity (Levine et al. 2014). These regulatory sequences occur in the context of nucleosomes, which provide a repressive ground state for gene expression (Struhl 1999; Wang et al. 2011). Nucleosome occupancy can be determined by multiple factors: DNA sequences, transcription factors, chromatin remodeling enzymes, and the transcriptional machinery (Struhl and Segal 2013). DNA sequences can favor or disfavor nucleosome formation. For example, sequences with poly(dA:dT) tracts are known to destabilize nucleosomes (Anderson and Widom 2001), whereas sequences with high GC content or ∼10-bp periodicities of AA or TT dinucleotides favor nucleosome formation (Ioshikhes et al. 2006; Segal et al. 2006; Tillo and Hughes 2009). In higher eukaryotes, the DNA sequence at promoters, enhancers, and transcription factor-binding sites generally encode a high intrinsic nucleosome occupancy (Tillo et al. 2010; Gaffney et al. 2012). However, in disagreement with predicted high nucleosome occupancy, genome-wide mapping typically shows that nucleosomes are depleted at active promoters (Schones et al. 2008; Valouev et al. 2011; Teif et al. 2012). DNA accessibility studies further indicate that promoters tend to open ubiquitously among multiple cell types (Thurman et al. 2012), suggesting that generally expressed *trans*-factors keep promoters accessible. However, enhancers tend to become open in a tissue-specific manner (Thurman et al. 2012).

How do enhancer chromatin sites become accessible in a tissue-specific manner against an intrinsic propensity of the underlying DNA for high nucleosome occupancy? Although most transcription factors cannot access nucleosomal DNA by themselves, pioneer transcription factors (e.g., FoxA) can bind to nucleosomal DNA and recruit other factors to bind (Cirillo et al. 2002). Along these lines, the transcription factors p53 and PU.1 and the progesterone receptor, which apparently operates with FoxA1 (Clarke and Graham 2012), preferentially target their binding motifs amid sequences with high intrinsic nucleosome occupancy rather than high-affinity motifs with low intrinsic nucleosome occupancy (Lidor Nili et al. 2010; Ballare et al. 2013; Barozzi et al. 2014). Thus, contrary to the dogma that nucleosomes would be inherently repressive to gene activity and must be removed for factor binding, pioneer transcription factors positively use the feature of high nucleosome occupancy at enhancers as their functional binding target (Fig. 2A).

Given that pioneer factors can bind their targets on nucleosomal DNA, where other factors cannot, we suggest that, at enhancers involved with cell and tissue identity, nucleosomes act as a filter to prevent binding of nonpioneer factors that prefer free DNA and would otherwise perturb a cell identity network. By this logic, if the target sequence became stably nucleosome-free, the nucleosome could no longer serve as such a filter. We need further studies of nucleosome stability at higher eukaryotic enhancers to assess these issues.

## Facilitators and impediments for pioneer factor binding in vivo

Genome-wide mapping studies show that all transcription factors, including pioneer factors, bind to only a subset of their putative binding motifs in the genome and show distinct binding patterns in different cell types. As an example of the requisite analysis, see the application of the computational tool MultiGPS to compare ChIP-seq data sets (Mahony et al. 2014). The observations of Mahony et al. (2014) led to the hypothesis that (1) pioneer factors can initiate targeting of a region and then engage other factors to stabilize a pioneer factor’s binding, (2) positive chromatin features can enable pioneer factor binding, and (3) repressive chromatin features can prevent pioneer factor binding. We discuss these possibilities below.

### Nucleosome-mediated cooperativity between transcription factors

The binding of multiple transcription factors that function combinatorially is inherent to eukaryotic gene regulation, particularly for cell-specific control (Yamamoto 1985; Carey 1998). As described earlier, transdifferentiation events typically require sets of transcription factors. In liver development, the pioneer factors FoxA and GATAs initially occupy a liver-specific enhancer (Gualdi et al. 1996). In macrophage and B-cell development, PU.1 acts as a pioneer transcription factor but requires a distinct set of lineage-determining transcription factors to enhance PU.1 binding at target sites and the deposition of active histone modifications (Heinz et al. 2010). Furthermore, in the initiation of iPSC reprogramming, Oct3/4, Sox2, and Klf4 act as pioneer transcription factors, and c-Myc facilitates Oct3/4, Sox2, and Klf4 binding (Soufi et al. 2012). These results suggest that pioneer transcription factors can bind coordinately with other factors. We speculate that the intrinsic nucleosome recognition properties of pioneer factors allows them to scan the closed chromatin for potential target sites and then recruit other factors, which could in turn stabilize the pioneer factor’s binding to chromatin (Fig. 2).

The most commonly known cooperative binding is based on protein–protein interaction between transcription factors. However, the nucleosome structure of DNA enables cooperative binding without such protein–protein interactions; that is, nucleosome-mediated cooperativity between transcription factors can be achieved by their simultaneous competition with histones for the underlying DNA (Adams and Workman 1995; Chavez and Beato 1997; Vashee et al. 1998; Miller and Widom 2003; Mirny 2010; Moyle-Heyrman et al. 2011). Such cooperativity typically can be seen when multiple (e.g., four to five) transcription factor-binding events can occur within one or one-half of a nucleosome. In contrast, pioneer factors on their own can recognize nucleosomal target sites and then recruit other transcription factors. Such recruitments could stabilize the binding of a pioneer and a nonpioneer factor to elicit a cell type-specific binding pattern. With or without such stabilization, the enhanced ability of pioneer factors to interact with nucleosomal DNA provides an advantage for cooperative mechanisms to facilitate the binding of multiple transcription factors to closed chromatin at silent genes.

### Positive chromatin features for pioneer factor binding

Do pioneer transcription factors positively use chromatin modifications to enhance their interaction with certain target sites? Here the data are mixed. First, recent studies indicate that ∼40% of the mammalian and *Droshophila* genomes lack distinctive histone modifications and are considered to be in a “low signal” state (Kharchenko et al. 2011; Ho et al. 2014). Thus, other than being nucleosomal, a large fraction of chromatin appears to be neutral—not marked for either activation or repression. Consistent with such observations, human Oct3/4, Sox2, and Klf4 in initial reprogramming bind to genomic sites that are not enriched, on average, for histone modifications (Soufi et al. 2012), and PU.1 binding does not depend on H3K4me1 but is followed by H3K4me1 deposition (Ghisletti et al. 2010; Heinz et al. 2010).

Two independent studies show that the deposition of active histone modifications (H3K4me2, H3K4me1, and H3K9ac) follow or are concomitant with FoxA binding but are not prior to FoxA binding during differentiation induced by retinoic acid in pluripotent ES or P19 embryonal carcinoma cells (Taube et al. 2010; Serandour et al. 2011). However, another study showed that reduction of H3K4me1 and H3K4me2 impaired FoxA binding in the breast cancer cell line MCF7 (Lupien et al. 2008). Altogether, it appears that active histone modifications may enhance pioneer factor binding to chromatin but are not necessarily required for the factor’s initial engagement. Also, DNA hypomethylation is not required for FoxA binding (Serandour et al. 2011). In terms of histone variants, FoxA2-binding sites can be preoccupied by H2A.Z during ES cell differentiation to endoderm/hepatic progenitor cells, but these preoccupied sites are only a fraction (16%) of the total FoxA2-binding sites (Li et al. 2012a). At this point, the most consistent chromatin feature that predicts engagement of pioneer transcription factors is high intrinsic nucleosome occupancy (Fig. 2).

### Repressive chromatin features for pioneer factor binding

Are there chromatin features that prevent a pioneer factor’s binding to nonfunctional or alternative lineage sites? Comparing between MCF7 breast cancer and LNCaP prostate cancer cell lines, FoxA1 binds differentially to more than half of its target sites (Lupien et al. 2008). This cell type-specific binding is inversely correlated with the repressive histone modification H3K9me2. Furthermore, megabase-scale heterochromatic domains spanned by H3K9me3 in fibroblasts contain genes required for cell reprogramming to pluripotent cells but are refractory to the initial binding by OSKM during the reprogramming (Fig. 1; Soufi et al. 2012). A reduction of H3K9me3 deposition allows Oct3/4 and Sox2 binding to these domains and enhances reprogramming. Strikingly, various genes that function late in the reprogramming process, causing reprogramming to proceed more deterministically in the cell population, map to the heterochromatic domains that are resistant to the initial OSKM binding (Buganim et al. 2012). It thus appears that the H3K9me3 heterochromatic domains represent a barrier to the conversion from the stochastic phase to the deterministic phase of reprogramming. Altogether, H3K9me2 and H3K9me3 domains seem to block pioneer factor binding.

Heterochromatic blocks to pioneer factor binding may provide a means for cells to stably retain their fate. We suggest that a reason why transdifferentiation typically fails to shut off an initial genetic program may relate to the failure of resetting heterochromatic domains for new cell type (Cahan et al. 2014). Understanding how heterochromatic regions are established and maintained will continue to provide insight into enhancing cell conversions and may help explain changes in cell fate in diseases such as cancer (Cruickshanks et al. 2013).

## Pioneer factors as bookmarking factors in mitosis

Pioneer transcription factors play an important role in cellular memory during mitotic growth. During mitosis, the chromosomes markedly condense, RNA polymerase is excluded from the chromatin, and transcription ceases. Recent chromosome conformation capture studies show that the three-dimensional partitioning of genomic domains in interphase is almost completely lost during mitosis (Naumova et al. 2013). During mitotic exit, such domains are re-established, RNA polymerase re-engages the chromatin, and the entire genome becomes transcriptionally activated in a manner that faithfully recapitulates the prereplicated state of the cell (Egli et al. 2008). Although most transcription factors and RNA polymerase II dissociate from mitotic chromatin, a subset of transcription factors are retained, originally referred to as “bookmarking” factors (Martinez-Balbas et al. 1995; Michelotti et al. 1997; Zaidi et al. 2010). In the time since this initial discovery, it has become appreciated that the mitotic-retaining bookmarking factors are typically those classified as pioneer transcription factors, including GATA1 and FoxA1 (Table 1; Kadauke et al. 2012; Caravaca et al. 2013). Genome-wide binding studies of GATA1 and FoxA1 in mitosis show that only a minority of their interphase binding sites are retained in mitotic chromatin (Kadauke et al. 2012; Caravaca et al. 2013). Interestingly, the mitotic-retained sites of binding in GATA1 and FoxA1 are not associated with a particular histone modification. The one correlate that was observed is that the mitotic-retained FoxA1 sites are those with a higher predicted intrinsic nucleosome occupancy (Caravaca et al. 2013). This is consistent with the above discussion about how predicted nucleosome occupancy is a prominent feature at genomic regulatory sequences and underscores the nucleosome-binding pioneer feature for mitotic bookmarking.

Notably, the genes associated with GATA1 and FoxA1 binding in mitosis were among the first to be reactivated during mitotic exit (Kadauke et al. 2012; Caravaca et al. 2013). A careful experiment in which GATA1 was destroyed specifically in mitosis proved definitively that mitotic retention was crucial for timely gene reactivation during mitotic exit (Kadauke et al. 2012). All of the known bookmarking/pioneer factors function in cell programming during development, leading to the suggestion that the mechanism of gene reactivation during mitotic exit could recapitulate, in part, the hierarchical processes by which different cell types are specified in the first place (Zaret 2014).

## Pioneer factors in disease progression

Since pioneer transcription factors so markedly affect cell reprogramming, their misregulation could cause severe effects on human health. *Sox2* is not expressed in the normal epidermis but is up-regulated in mouse and human cancer stem cells in skin squamous cell carcinomas (Boumahdi et al. 2014). There, Sox2 is involved in the initial stage of tumor formation and its maintenance. In human esophageal and lung squamous cell carcinomas, chromosome segments containing *Sox2* are often genetically amplified (Bass et al. 2009). Thus, ectopic Sox2 expression or a higher level of its expression could activate previously silent programs to establish competence for tumorigenesis, perhaps analogous to cell reprogramming. Furthermore, FoxA and GATA factors are involved in a variety of hormone-dependent cancers, such as estrogen receptor-positive breast cancer and androgen receptor-positive prostate cancer (Zaret and Carroll 2011; Jozwik and Carroll 2012). In breast cancer, FoxA1 expression is a significant predictor of cancer-specific survival in patients (Badve et al. 2007). Thus, pioneer transcription factors would be potential biomarkers and drug targets for cancers treatment.

## Pioneer factors and the future of cellular reprogramming

As seen from the above discussion, pioneer factors are distinguished by their ability to recognize target DNA sequences on nucleosomes under conditions where other factors cannot. Such binding may be stabilized by coordinate actions of other proteins and is typically succeeded by changes in the local chromatin structure, thereby enabling subsequent events required for gene regulation. The ability to target silent genes embedded in closed chromatin makes pioneer factors especially suitable for cellular reprogramming, much as they have evolved for their natural roles in cellular programming in development. Still, important questions remain (Box 2).

**Box 2. Important issues to be addressed**

What is the influence of predicted high nucleosome occupancy and nucleosome position on defining the subset of target sites to which pioneer factors will bind in a cell?
What are the structural features of DNA-binding domains that allow certain factors to have pioneer activity?
How do domains outside of the DNA-binding domain affect pioneer activity and subsequent chromatin-opening events?
How does a better understanding of the mechanism of pioneer factor interaction with nucleosomes or chromatin inform how to generate transcription factors with enhanced cellular reprogramming activity?
How do chromatin features (e.g., H3K9me3 domains) impede pioneer factor binding?
How are heterochromatic domains established or broken down in order to impede or enhance pioneer factor binding?

Despite their special nucleosome-binding ability, how is it that pioneer factors recognize only a subset of their target sites in native chromatin? As we discussed here, perhaps the subset of sites that are targeted by pioneer factors involve ensembles of positioned nucleosomes in a particular cell type that favor pioneer binding. More detailed mapping of nucleosome positions in cells, along with how they may or may not be targeted by transcription factors, should provide insight.

How do different DNA-binding domain structures enable or inhibit pioneer activity? This question seems most efficiently addressed by detailed biochemical studies, comparing how different pioneer and nonpioneer factors bind (or not) to purified, defined nucleosomes. Are there preferred motif variants for targeting a closed chromatin site in the genome versus open sites, and how does the ability of a DNA-binding domain to adapt to nucleosomal DNA relate to nucleosome-binding capacity? This could be modeled on nucleosome substrates in vitro and the information used to modify a DNA-binding domain in order to enhance its initial targeting to nucleosomal DNA, thereby enhancing cellular reprogramming. This strategy is feasible because the transcription factors that are used to elicit cellular reprogramming appear to be needed only for the initial activation of the appropriate cellular networks. After initiation, the exogenous reprogramming factors can be extinguished, while the new networks self-sustain and complete the fate conversion in the absence of the initial reprogramming factors (Takahashi and Yamanaka 2006).

How might pioneer protein domains outside of the DNA-binding domain affect nucleosome architecture and stability? Other protein domains could interact with the core histones to stabilize nucleosome binding. Furthermore, it remains to be determined whether pioneer factors “tickle” the nucleosome in a way that destabilizes the core histones or changes nucleosome conformation in a way that modulates how other factors can bind. In addition, it will be important to perform detailed biochemical studies on polynucleosomal templates so that protein domains that affect higher-order folding of the local chromatin can be assessed (Cirillo et al. 2002). Conceivably, such chromatin-opening domains could be transferred to other factors and augment their regulatory function.

Exactly how does highly compacted heterochromatin exclude pioneer factor access? Understanding how tissue-specific heterochromatic domains are made and can be broken down will provide much insight into ways to enhance cellular reprogramming. Assuming that the terminal establishment of a new cell fate after reprogramming will involve the resetting of heterochromatic domains, it seems likely that transient rather than permanent methods for heterochromatin diminution at the onset of cellular reprogramming will be the most useful.

It is presently possible to use small molecules to target chromatin modifications that globally affect most cell networks at once. We anticipate that understanding the mechanisms by which pioneer factors target specific sites in the genome, initiate regulatory events, and maintain them through mitosis and other contexts will provide more specific means for modulating cell networks that control cell fate and function in health and disease.
